# Custom Nasal Stent Fabrication for Post-traumatic Nasal Obstruction - A Case Report

**DOI:** 10.7759/cureus.31843

**Published:** 2022-11-23

**Authors:** Rama Shankar, Mridu Dubey

**Affiliations:** 1 Dentistry, Tata Main Hospital, Jamshedpur, IND

**Keywords:** nasal obstruction, nasal valve narrowing, nasal stent, alar collapse, nostril stenosis

## Abstract

Deformities of the nose can be of congenital, traumatic, or iatrogenic etiology. The aim of treating nasal/nostril stenosis is to establish an adequate airway and restore facial profile; this may be achieved by surgical correction or prosthetic means. This case report presents the fabrication of a customized rigid acrylic stent for alar stenosis in an 18-year-old girl occurring as a result of trauma. The functional and aesthetic result is evaluated at six months. Thus, customized acrylic nasal stents provide an easy-to-fabricate, economical option for the maintenance of nasal contour and patency of the airway.

## Introduction

The nose is the most prominent feature of the human face, comprising an osseocartilaginous framework and supporting soft tissues. Nostril, or the naris, is the entrance to the air passage, and stenosis of the nostril may occur secondary to infection, trauma, or iatrogenic insult, with consequent mouth breathing, patient discomfort, and aesthetic concern [1]. The external nasal valve area, located in the area of nostrils, is formed by the lower lateral cartilage, the soft tissue ala and medially, the columella and septum [2]. Any injury to the fibro-fatty or fibromuscular tissue of the ala may potentially result in its narrowing and flattening. The goal of reconstruction/rehabilitation in patients with alar collapse is to emulate the anatomy and restore form and function. Different surgical techniques have been described for the correction of nostril stenosis, including the use of flaps and grafts [3,4].

However, surgical complications resulting in re-stenosis are well described in literature. Thus, prosthetic support of cartilage-deficient nasal tissue is essential to preserve the contour of the nostril during wound healing, maintain airway patency and enhance aesthetics [5-8]. The purpose of this case report was to present a case of unilateral post-traumatic alar collapse managed with a customized nasal stent.

## Case presentation

**Figure 1:**
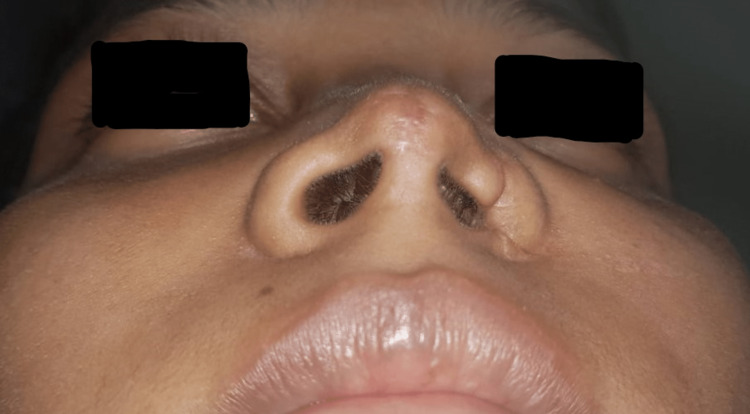
Collapse of the left ala

An 18-year-old female was referred from the otorhinolaryngology department to the department of dentistry of Tata Main Hospital for the fabrication of a nasal stent prior to reconstructive surgery. The patient had a history of trauma due to a road traffic accident two weeks ago with a lacerated wound in the region of the left ala, which was sutured in the emergency. She reported to the ENT department with a complaint of discomfort in breathing, with the left nose collapsing inward during inspiration (Figure 1). Examination revealed narrowing of the ala of the left nose as a consequence of wound healing.

A customized nasal stent was then planned to provide support to the collapsed ala while maintaining airway patency and allowing for acceptable aesthetics. The goal was to obtain adequate correction in a conservative manner in order to minimize the need for surgical intervention.

**Figure 2:**
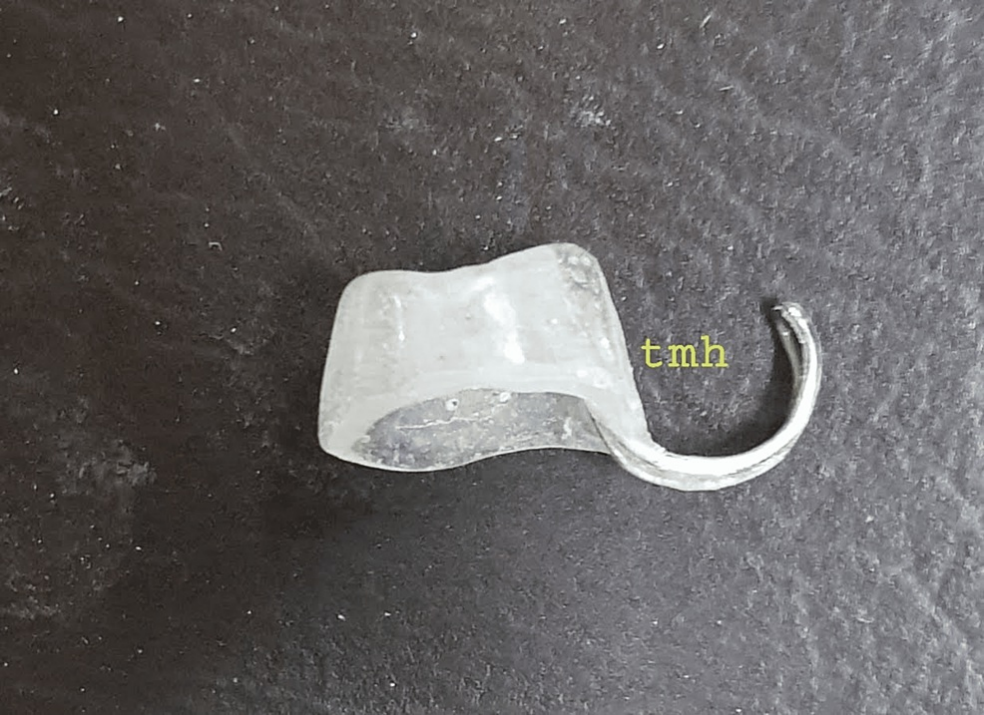
Custom nasal prosthesis processed in clear acrylic resin with a wire loop TMH - Tata Main Hospital

An impression of the entire nose was made using irreversible hydrocolloid impression material, following lubrication of the nasal cavities with petroleum jelly. The patient was seated upright and advised to breathe through the mouth during the duration of the impression-making procedure. The impression was cast in die stone (type IV) and processed in clear acrylic resin (Figure 2).

**Figure 3:**
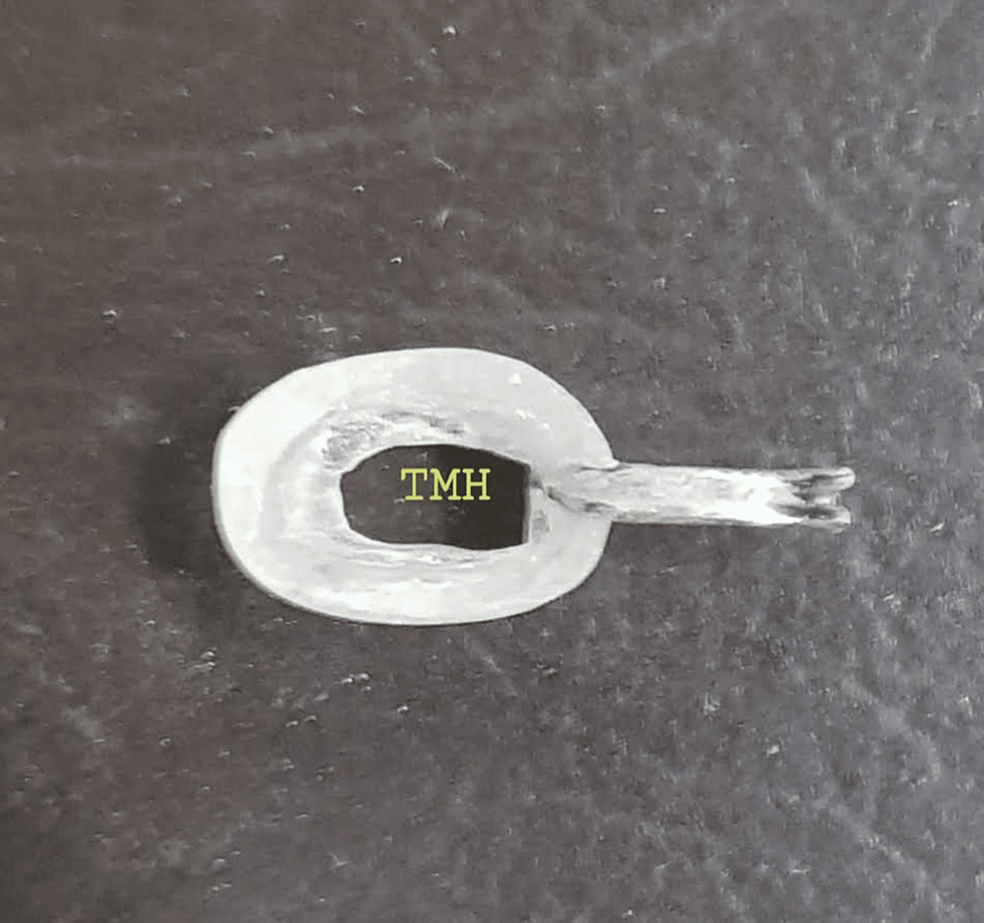
Pinhole drilled in the prosthesis to provide an airway TMH - Tata Main Hospital

**Figure 4:**
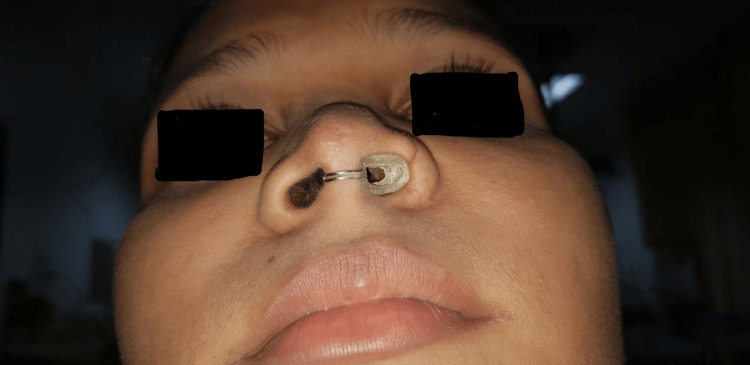
Intranasal prosthesis in-situ

A stent that is a mirror image of the contralateral nostril was believed to lead to an adequate opening. A wire loop that would engage across the columella was embedded in the pattern to provide retention. A 4mm hole was drilled through the finished prosthesis to provide an airway (Figure 3). The fit of the prosthesis was evaluated for aesthetics and comfort (Figure 4).

**Figure 5:**
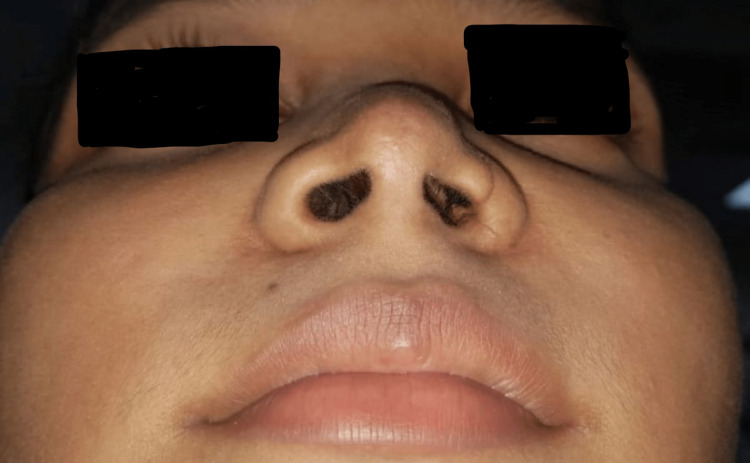
Six month follow-up

The patient was advised to wear the prosthesis continuously and to remove it only for daily cleaning with soap and water. She was evaluated at the three month and six month intervals. At the three month follow-up appointment, the patient could breathe comfortably, and the aesthetics were acceptable. Thus, she was advised only night-time use of the stent until the next review at six months (Figure 5).

## Discussion

Stenosis of the nasal vestibule can occur due to variable aetiology. Acquired causes like trauma, infection, and iatrogenic insult are more common than congenital causes [9,10]. Trauma may involve direct injury to the ala or damage to the vestibular lining. Since most of the ala is composed of fibrofatty or fibromuscular tissue, scar formation during the healing phase after an injury may contract and flatten the ala [11].

The goal of treating the post-traumatic nose involves restoring the form and function of the nose [12]. While various flaps and grafts [13-15] have been proposed to replace the lost tissue, these surgical procedures may result in further stenosis. Thus, a nostril stent is often recommended post-operatively to maintain the corrected position of the nose [16].

In the presented case, the patient has developed alar collapse, exacerbated by the negative pressure during inspiration, as a result of scar formation following the healing of the sutured lacerated wound on the left ala after a road traffic accident. She was referred to the department of dentistry for the fabrication of a stent to support the soft tissue, re-establish an adequate airway and improve the cosmetic appearance while the patient awaited definitive reconstructive surgery. The alar stent is also believed to act as a device for pre-surgical alignment and correction by expanding the tissues and leading to more consistent postoperative outcomes [17].

Customized stents may be fabricated with acrylic resin or silicone. Hard/rigid acrylic resin stents have the advantage of being easy to fabricate, are economical, and lend themselves to modification post-processing. Meanwhile, soft silicone stents provide greater comfort and can adapt to undercuts but cannot be modified after processing. In addition, they are believed to be more susceptible to fungal growth [5]. In a study conducted to assess the 24-month outcome of customized alar stents, the authors found that rigid nasal dilators were associated with a more positive and faster improvement result as compared to soft dilators [17].

Stents placed in-situ immediately post-op may be held in place with sutures or held in place by an adhesive tape across the inferior border of the nose [1]. In this case, a wire loop was used for retention, which was more comfortable and aesthetically mimicked an ornamental nose ring. The importance of an extremely smooth, polished surface of the prosthesis cannot be overemphasized, to prevent damage to the nasal mucosa during repeated insertion and removal.

Different period of stent use has been advocated in literature depending on the degree of narrowing and the age of the patient, ranging from six weeks to a year [5,11]. In the reported case, the patient wore the stent continuously for a period of three months, after which she discontinued the use with no effect on the outcome at the six month follow-up. This may be attributed to the fact that sufficient fibrosis and consolidation of the tissues would have been achieved.

## Conclusions

The use of a customized alar stent provides a comfortable, economic, and minimally invasive approach to the management of post-traumatic nasal stenosis, especially when the obstruction is in the anterior portion of the nasal passage. Such a prosthesis is easy to fabricate and facilitates breathing with acceptable aesthetics while minimizing post-healing cicatrization.
